# Crystal structure of *catena*-poly[[potassium-tri-μ-di­methyl­acetamide-κ^6^ *O*:*O*] iodide]

**DOI:** 10.1107/S1600536814020005

**Published:** 2014-09-13

**Authors:** Cezar-Catalin Comanescu, Allen G. Oliver

**Affiliations:** a Department of Chemistry and Biochemistry, University of Notre Dame, Notre Dame, IN 46556-5670, USA

**Keywords:** crystal structure, one-dimensional coordination polymer, symmetry, di­methyl­acetamide, potassium salt

## Abstract

The structure of *catena*-poly[[potassium-tri-μ-di­methyl­acetamide-κ^6^ *O*:*O*] iodide], {[K(C_4_H_9_NO)_3_]I}_*n*_, at 120 K has trigonal (*P*-3) symmetry. The structure adopts a linear chain motif parallel to the crystallographic *c* axis. Two crystallographically independent K^+^ cations are present in the asymmetric unit located on threefold rotoinversion axes at [0, 0, 0] and [0, 0, 1/2] and are bridged by the O atoms of the acetamide moiety. This is an example of a rare μ_2_-bridging mode for di­methyl­acetamide O atoms. The iodide counter-ion resides on a threefold rotation axis in the channel formed by the [K(C_4_H_9_NO)]^+^ chains.

## Abstract 2

A one-dimensional chain formed by potassium bridged by μ_2_-*O*-di­methyl­acetamide is reported. This represents a small class of di­methyl­acetamide complexes with oxygen bridging two metal centers.

## Chemical context   

Coordination of di­methyl­acetamide (DMA) to metal centers has been observed previously in a number of metal complexes, but μ_2_-coordination of the O atom has only been reported in two crystallographically confirmed structures. Tikhonova *et al.* (2001 ▶) crystallized a bis­(μ_3_-*N*,*N*-di­methyl­acetamide)­tris­(μ_2_-perfluoro-*o*-phenyl­ene)trimercury(II) complex and found Hg—O(DMA) bond lengths in the range 2.776 (2)–2.989 (2) Å. Dias *et al.* (1995 ▶) synthesized bis­{(μ_2_-di­methyl­acetamido-*O*,*O*){μ_2_-hydrogen tris­[3,5-bis­(tri­fluoro­meth­yl)pyra­zol­yl]borate}potassium}, in which the O atom is μ_2_-bridging between two K^+^ cations and the K—O bond length is 2.703 (2) Å. In the KI·3DMA structure reported here, the K—O bond lengths are in the range 2.763 (2)–2.774 (3) Å, slightly longer than in the closely related potassium complex synthesized by Dias *et al.* (1995 ▶).
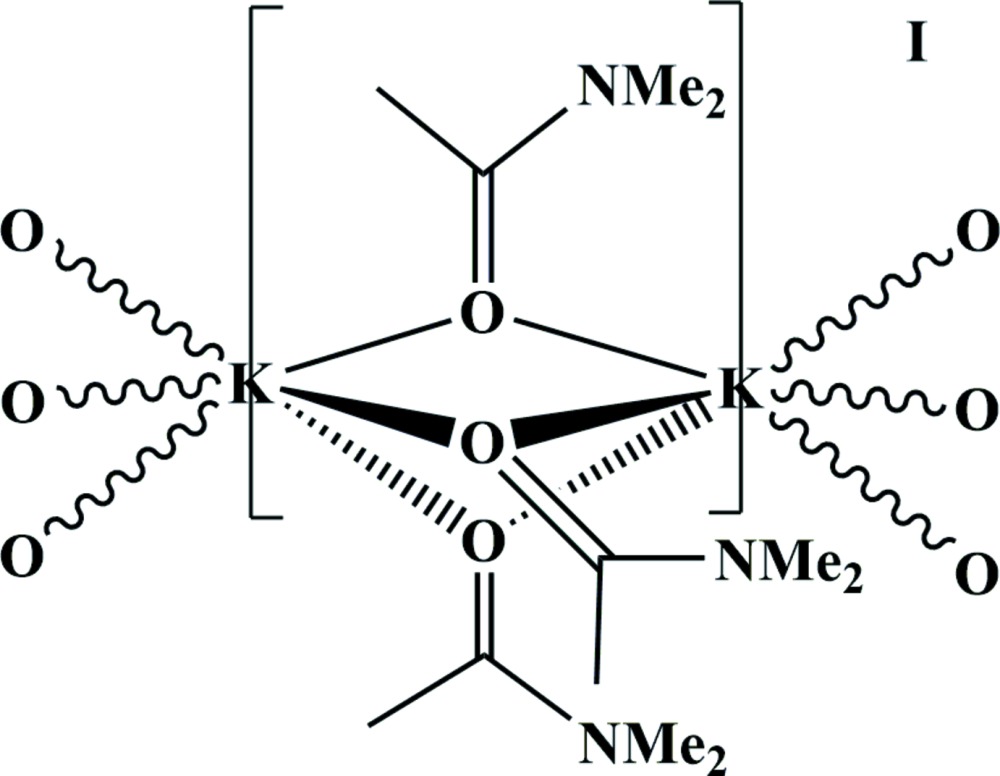


## Structural commentary   

**Figure 1:**
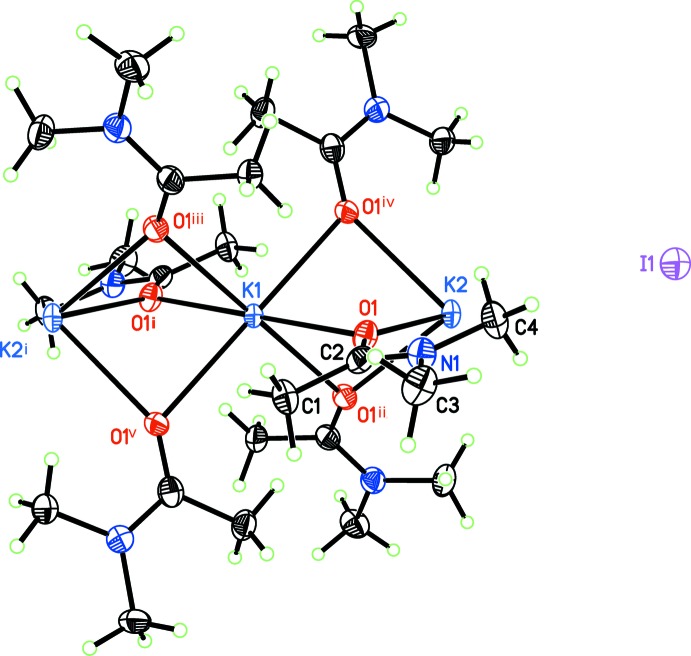
The atom-labeling scheme for KI·3DMA, with displacement ellipsoids depicted at the 50% probability level. [Symmetry codes: (i) −*x*, −*y*, −*z*; (ii) *y*, −*x* + *y*, −*z*; (iii) −*y*, *x* − *y*, *z*; (iv) *x* − *y*, *x*, −*z*; (v) −*x* + *y*, −*x*, *z*; (vi) *x*, *y*, *z* − 1.]

The cation of title compound consists of two crystallographically independent potassium cations. Each K^+^ cation is octa­hedrally coordinated by six O atoms from the DMA moieties, with each oxygen adopting a μ_2_-bridging mode (Fig. 1 ▶ and Table 1 ▶). The C=O distance is comparable with that in free di­methyl­acetamide (see *Database survey* [Sec sec4]). The iodide anion is independent of the one-dimensional chain and does not form any covalent contacts to the cation.

The extended structure forms a chain of K^+^ cations, bridged by μ_2_-*O*-di­methyl­acetamide moieties. The two independent K^+^ cations are located at [0, 0, 0] and [0, 0, ½] (Wyckoff positions *a* and *b*, respectively) and the iodine is located at [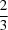, 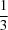, z] (Wyckoff position *d*). In the primary structure, each K^+^ cation adopts a slightly distorted octa­hedral coordination sphere (key bond lengths and angles are given in Table 1 ▶).

## Supra­molecular features   

**Figure 2:**
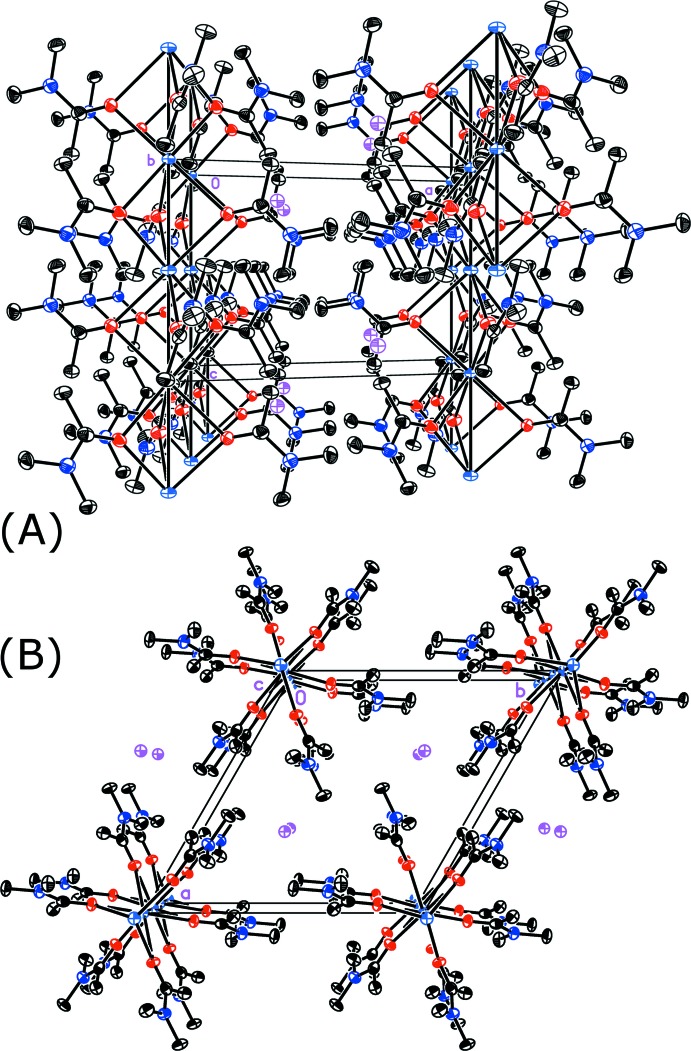
(*A*) Packing diagram viewed along the *b* axis. (*B*) View along the *c* axis. Legend: black = carbon, dark blue = nitro­gen, light blue = potassium, magenta = iodine, and red = oxygen. H atoms have been omitted for clarity.

The μ_2_-*O*-di­methyl­acetamide bridging the two K^+^ cations forms a linear [K(DMA)_3_]^+^ chain parallel to the *c* axis. The application of the 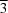 symmetry results in an aesthetic­ally pleasing ‘snowflake’ configuration when viewed along the *c* axis (Fig. 2 ▶). The iodide counter-ion resides in the channels formed by the [K(DMA)_3_]^+^ chains. With regards to the extended structure, there are very weak C—H⋯I inter­actions within the lattice (Table 2 ▶). These serve to locate the iodine in a pocket within the structure.

## Database survey   

A search in the Cambridge Structure Database (CSD, Version 5.35, November 2013 plus three updates; Allen, 2002 ▶) for structures in which K^+^ is triple bridged in a μ_2_-fashion by three O atoms returns 17 results, but only 3 of them are relevant to the structure reported herein. Gonzalez-Rodriguez *et al.* (2009 ▶) have shown a complex guanosine-derived nucleoside to crystallize as an acetone solvate monohydrate in which the six bridging K^+^ cations are each coordinated to eight O atoms from eight guanosine ligands, and the two terminal K^+^ cations are coordinated to eight O atoms from four guanosine ligands and either four acetone mol­ecules or four water mol­ecules. Cunningham *et al.* (2000 ▶) crystallized *catena*-[tetra­kis­[*N*,*N*′-bis­(3-meth­oxy­salicyl­idene)propane-1,3-di­amino­ato]iodido­nickel(II)potassium], where K^+^ is bridged by four μ_2_-*O*, one μ_2_-*N*, and one μ_2_-*I*. In fact, both of these structures contain four μ_2_-*O* atoms bridging K^+^ cations. No close K⋯K contacts were observed: the K⋯K distances are in the range 3.451 (2)–3.567 (2) Å. Most closely related is the structure of *catena*-[tris­(μ_2_-di­methyl­formamide-*O*,*O*)potassium iodide], reported by Batsanov & Struchkov (1994 ▶), with a K⋯K distance of 3.4170 (10) Å and a K—O distance of 2.6570 (13) Å. In the KI·3DMA structure reported herein, the K1⋯K2 distance is 3.6728 (4) Å, which is longer by approximately 0.106 Å. In the Gonzalez-Rodriguez and Cunningham structures, iodine is found to form bonds to the K^+^ cations, while it is located in a channel within the Batsanov structure and not covalently bound. In the title compound, the iodine is not covalently bonded to the cation chain.

A search in the Cambridge Structure Database for free acetamide returned 180 results, featuring C=O bond lengths between 1.123 Å (Patra & Goldberg, 2013 ▶) and 1.67 Å (Gole *et al.*, 2011 ▶), with a mean of 1.259 Å (std. dev. 0.059), which is very close to the C=O bond length reported herein [1.254 (3) Å]

## Synthesis and crystallization   

A carbon–carbon Heck coupling reaction catalyzed by a Pd^II^ diphosphane precatalyst was performed using conditions established previously by Brase & de Meijere (1998 ▶). In a typical synthesis, 1-iodo-4-nitro­benzene (IC_6_H_4_NO_2_; 102.1 mg, 0.41 mmol) was mixed with 2 equivalents of *n*-butyl acrylate [CH_2_=CHCOO(CH_2_)_3_CH_3_; 105.6 mg, 0.82 mmol] in the presence of K_2_CO_3_ (63.6 mg, 0.46 mmol) and *n*-Bu_4_NBr (13 mg, 0.041 mmol) in di­methyl­acetamide (DMA) over a period of 4 h at 413 K. The title compound formed and was recrystallized from the filtered reaction mixture at room temperature. The target Pd^II^ complex of the reaction has been reported (Comanescu & Iluc, 2014 ▶).

## Refinement   

Crystal data, data collection and structure refinement details are summarized in Table 3 ▶. H atoms were included in a riding model and allowed to rotate to minimize electron-density contribution. C—H distances were set at 0.98 Å, with *U*_iso_(H) = 1.5*U*_eq_(C).

## Supplementary Material

Crystal structure: contains datablock(s) I. DOI: 10.1107/S1600536814020005/zl2601sup1.cif

Structure factors: contains datablock(s) I. DOI: 10.1107/S1600536814020005/zl2601Isup2.hkl

Supporting information file. DOI: 10.1107/S1600536814020005/zl2601Isup3.cml

CCDC reference: 1022963

Additional supporting information:  crystallographic information; 3D view; checkCIF report

## Figures and Tables

**Table 1:** Selected geometric parameters (Å, °)

K1—O1	2.7438 (16)	O1—C2	1.254 (3)
K1—K2	3.6728 (4)	O1—K2	2.7627 (16)
			
O1^i^—K1—O1	180.0	O1^iii^—K1—O1	80.70 (5)
O1^ii^—K1—O1	99.30 (5)		

Symmetry codes: (i) 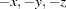; (ii) 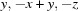; (iii) 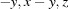.

**Table 2:** Hydrogen-bond geometry (Å, °)

*D*—H⋯*A*	*D*—H	H⋯*A*	*D*⋯*A*	*D*—H⋯*A*
C1—H1*A*⋯I1^iv^	0.98	3.20	4.178 (3)	177
C3—H3*A*⋯I1^iv^	0.98	3.20	4.178 (3)	174
C4—H4*C*⋯I1	0.98	3.00	3.967 (3)	170

Symmetry code: (iv) 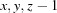.

**Table 3:** Experimental details

Crystal data	
Chemical formula	[K(C_4_H_9_NO)_3_]I
*M*_r_	427.37
Crystal system, space group	Trigonal, *P* 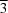
Temperature (K)	120
*a*, *c* (Å)	11.9776 (8), 7.3455 (7)
*V* (Å^3^)	912.62 (15)
*Z*	2
Radiation type	Mo *K*α
μ (mm^−1^)	1.99
Crystal size (mm)	0.20 × 0.09 × 0.06

Data collection	
Diffractometer	Bruker APEX
Absorption correction	Multi-scan (*SADABS*; Bruker, 2012 ▶)
*T*_min_, *T*_max_	0.615, 0.745
No. of measured, independent and observed [*I* > 2σ(*I*)] reflections	11940, 1248, 1194
*R*_int_	0.026
(sin θ/λ)_max_ (Å^−1^)	0.623

Refinement	
*R*[*F* ^2^ > 2σ(*F* ^2^)], *wR*(*F* ^2^), *S*	0.024, 0.059, 1.07
No. of reflections	1248
No. of parameters	65
H-atom treatment	H-atom parameters constrained
Δρ_max_, Δρ_min_ (e Å^−3^)	1.14, −0.43

Computer programs: *APEX2* and *SAINT* (Bruker, 2012 ▶), *SHELXS97*, *SHELXL2013* and *XP* in *SHELXTL* (Sheldrick, 2013 ▶) and *publCIF* (Westrip, 2010 ▶).

## supplementary crystallographic information

### Crystal data

**Table:**

[K(C_4_H_9_NO)_3_]I	*D*_x_ = 1.555 Mg m^−^^3^
*M**_r_* = 427.37	Mo *K*α radiation, λ = 0.71073 Å
Trigonal, *P*3	Cell parameters from 7529 reflections
*a* = 11.9776 (8) Å	θ = 2.7–26.3°
*c* = 7.3455 (7) Å	µ = 1.99 mm^−^^1^
*V* = 912.62 (15) Å^3^	*T* = 120 K
*Z* = 2	Block, colorless
*F*(000) = 432	0.20 × 0.09 × 0.06 mm

### Data collection

**Table:**

Bruker APEX diffractometer	1248 independent reflections
Radiation source: fine-focus sealed tube	1194 reflections with *I* > 2σ(*I*)
Graphite monochromator	*R*_int_ = 0.026
Detector resolution: 8.33 pixels mm^-1^	θ_max_ = 26.3°, θ_min_ = 2.0°
combination of ω and φ–scans	*h* = −14→14
Absorption correction: multi-scan (*SADABS*; Bruker, 2012)	*k* = −14→14
*T*_min_ = 0.615, *T*_max_ = 0.745	*l* = −9→9
11940 measured reflections	

### Refinement

**Table:**

Refinement on *F*^2^	Primary atom site location: structure-invariant direct methods
Least-squares matrix: full	Secondary atom site location: difference Fourier map
*R*[*F*^2^ > 2σ(*F*^2^)] = 0.024	Hydrogen site location: inferred from neighbouring sites
*wR*(*F*^2^) = 0.059	H-atom parameters constrained
*S* = 1.07	*w* = 1/[σ^2^(*F*_o_^2^) + (0.0227*P*)^2^ + 1.7319*P*] where *P* = (*F*_o_^2^ + 2*F*_c_^2^)/3
1248 reflections	(Δ/σ)_max_ = 0.001
65 parameters	Δρ_max_ = 1.14 e Å^−^^3^
0 restraints	Δρ_min_ = −0.43 e Å^−^^3^

### Special details

**Table:**

Geometry. All e.s.d.'s (except the e.s.d. in the dihedral angle between two l.s. planes) are estimated using the full covariance matrix. The cell e.s.d.'s are taken into account individually in the estimation of e.s.d.'s in distances, angles and torsion angles; correlations between e.s.d.'s in cell parameters are only used when they are defined by crystal symmetry. An approximate (isotropic) treatment of cell e.s.d.'s is used for estimating e.s.d.'s involving l.s. planes.

### Fractional atomic coordinates and isotropic or equivalent isotropic displacement parameters (Å^2^)

**Table:**

	*x*	*y*	*z*	*U*_iso_*/*U*_eq_	
I1	0.6667	0.3333	0.83886 (4)	0.02490 (11)	
K1	0.0000	0.0000	0.0000	0.0172 (2)	
O1	0.18933 (16)	0.14411 (16)	0.2481 (2)	0.0229 (4)	
N1	0.3896 (2)	0.2882 (2)	0.3421 (3)	0.0262 (5)	
C1	0.3288 (3)	0.3114 (3)	0.0349 (3)	0.0281 (5)	
H1A	0.4093	0.3202	−0.0125	0.042*	
H1B	0.3388	0.3971	0.0517	0.042*	
H1C	0.2588	0.2620	−0.0516	0.042*	
K2	0.0000	0.0000	0.5000	0.0227 (3)	
C2	0.2970 (2)	0.2414 (2)	0.2163 (3)	0.0249 (5)	
C3	0.5155 (3)	0.4036 (3)	0.3094 (4)	0.0320 (6)	
H3A	0.5574	0.3884	0.2054	0.048*	
H3B	0.5697	0.4223	0.4178	0.048*	
H3C	0.5033	0.4770	0.2830	0.048*	
C4	0.3621 (3)	0.2247 (3)	0.5200 (4)	0.0315 (6)	
H4A	0.3164	0.1310	0.5031	0.047*	
H4B	0.3083	0.2488	0.5913	0.047*	
H4C	0.4432	0.2516	0.5848	0.047*	

### Atomic displacement parameters (Å^2^)

**Table:**

	*U*^11^	*U*^22^	*U*^33^	*U*^12^	*U*^13^	*U*^23^
I1	0.02578 (13)	0.02578 (13)	0.02315 (16)	0.01289 (6)	0.000	0.000
K1	0.0195 (4)	0.0195 (4)	0.0126 (5)	0.00975 (18)	0.000	0.000
O1	0.0183 (8)	0.0237 (9)	0.0202 (8)	0.0057 (7)	−0.0003 (6)	−0.0034 (7)
N1	0.0242 (11)	0.0271 (11)	0.0237 (10)	0.0100 (9)	0.0003 (8)	−0.0003 (8)
C1	0.0284 (13)	0.0352 (14)	0.0196 (12)	0.0152 (11)	0.0012 (10)	0.0047 (10)
K2	0.0278 (4)	0.0278 (4)	0.0123 (5)	0.0139 (2)	0.000	0.000
C2	0.0291 (13)	0.0286 (13)	0.0223 (12)	0.0185 (11)	0.0013 (10)	−0.0037 (10)
C3	0.0212 (12)	0.0280 (13)	0.0336 (14)	0.0025 (11)	0.0023 (10)	−0.0045 (11)
C4	0.0325 (14)	0.0321 (14)	0.0218 (12)	0.0100 (12)	−0.0043 (11)	0.0038 (10)

### Geometric parameters (Å, º)

**Table:**

K1—O1^i^	2.7437 (16)	C1—H1B	0.9800
K1—O1^ii^	2.7437 (16)	C1—H1C	0.9800
K1—O1^iii^	2.7437 (16)	K2—O1^vii^	2.7627 (16)
K1—O1^iv^	2.7437 (16)	K2—O1^v^	2.7627 (16)
K1—O1^v^	2.7437 (16)	K2—O1^viii^	2.7627 (16)
K1—O1	2.7438 (16)	K2—O1^iii^	2.7627 (16)
K1—K2^vi^	3.6728 (4)	K2—O1^ix^	2.7627 (17)
K1—K2	3.6728 (4)	K2—K1^x^	3.6728 (4)
O1—C2	1.254 (3)	C3—H3A	0.9800
O1—K2	2.7627 (16)	C3—H3B	0.9800
N1—C2	1.333 (3)	C3—H3C	0.9800
N1—C4	1.465 (3)	C4—H4A	0.9800
N1—C3	1.468 (3)	C4—H4B	0.9800
C1—C2	1.517 (3)	C4—H4C	0.9800
C1—H1A	0.9800		
			
O1^i^—K1—O1^ii^	80.70 (5)	O1^v^—K2—O1^viii^	99.97 (5)
O1^i^—K1—O1^iii^	99.30 (5)	O1^vii^—K2—O1^iii^	99.97 (5)
O1^ii^—K1—O1^iii^	180.00 (7)	O1^v^—K2—O1^iii^	80.03 (5)
O1^i^—K1—O1^iv^	80.70 (5)	O1^viii^—K2—O1^iii^	180.0
O1^ii^—K1—O1^iv^	80.70 (5)	O1^vii^—K2—O1^ix^	80.03 (5)
O1^iii^—K1—O1^iv^	99.30 (5)	O1^v^—K2—O1^ix^	99.97 (5)
O1^i^—K1—O1^v^	99.30 (5)	O1^viii^—K2—O1^ix^	80.03 (5)
O1^ii^—K1—O1^v^	99.30 (5)	O1^iii^—K2—O1^ix^	99.97 (5)
O1^iii^—K1—O1^v^	80.70 (5)	O1^vii^—K2—O1	99.97 (5)
O1^iv^—K1—O1^v^	180.00 (7)	O1^v^—K2—O1	80.03 (5)
O1^i^—K1—O1	180.0	O1^viii^—K2—O1	99.96 (5)
O1^ii^—K1—O1	99.30 (5)	O1^iii^—K2—O1	80.04 (5)
O1^iii^—K1—O1	80.70 (5)	O1^ix^—K2—O1	180.0
O1^iv^—K1—O1	99.30 (5)	O1^vii^—K2—K1	132.06 (3)
O1^v^—K1—O1	80.70 (5)	O1^v^—K2—K1	47.94 (3)
O1^i^—K1—K2^vi^	48.39 (3)	O1^viii^—K2—K1	132.06 (3)
O1^ii^—K1—K2^vi^	48.39 (3)	O1^iii^—K2—K1	47.94 (3)
O1^iii^—K1—K2^vi^	131.61 (3)	O1^ix^—K2—K1	132.06 (3)
O1^iv^—K1—K2^vi^	48.39 (3)	O1—K2—K1	47.94 (3)
O1^v^—K1—K2^vi^	131.61 (3)	O1^vii^—K2—K1^x^	47.94 (3)
O1—K1—K2^vi^	131.61 (3)	O1^v^—K2—K1^x^	132.06 (3)
O1^i^—K1—K2	131.61 (3)	O1^viii^—K2—K1^x^	47.94 (3)
O1^ii^—K1—K2	131.61 (3)	O1^iii^—K2—K1^x^	132.06 (3)
O1^iii^—K1—K2	48.39 (3)	O1^ix^—K2—K1^x^	47.94 (3)
O1^iv^—K1—K2	131.61 (3)	O1—K2—K1^x^	132.06 (3)
O1^v^—K1—K2	48.39 (3)	K1—K2—K1^x^	180.0
O1—K1—K2	48.39 (3)	O1—C2—N1	121.0 (2)
K2^vi^—K1—K2	180.0	O1—C2—C1	122.3 (2)
C2—O1—K1	127.11 (15)	N1—C2—C1	116.8 (2)
C2—O1—K2	147.87 (15)	N1—C3—H3A	109.5
K1—O1—K2	83.67 (5)	N1—C3—H3B	109.5
C2—N1—C4	118.5 (2)	H3A—C3—H3B	109.5
C2—N1—C3	121.9 (2)	N1—C3—H3C	109.5
C4—N1—C3	119.6 (2)	H3A—C3—H3C	109.5
C2—C1—H1A	109.5	H3B—C3—H3C	109.5
C2—C1—H1B	109.5	N1—C4—H4A	109.5
H1A—C1—H1B	109.5	N1—C4—H4B	109.5
C2—C1—H1C	109.5	H4A—C4—H4B	109.5
H1A—C1—H1C	109.5	N1—C4—H4C	109.5
H1B—C1—H1C	109.5	H4A—C4—H4C	109.5
O1^vii^—K2—O1^v^	180.0	H4B—C4—H4C	109.5
O1^vii^—K2—O1^viii^	80.03 (5)		
			
K1—O1—C2—N1	−167.19 (17)	C4—N1—C2—O1	−1.0 (4)
K2—O1—C2—N1	32.0 (4)	C3—N1—C2—O1	−178.7 (2)
K1—O1—C2—C1	12.8 (3)	C4—N1—C2—C1	179.0 (2)
K2—O1—C2—C1	−148.1 (2)	C3—N1—C2—C1	1.4 (4)

Symmetry codes: (i) −*x*, −*y*, −*z*; (ii) *y*, −*x*+*y*, −*z*; (iii) −*y*, *x*−*y*, *z*; (iv) *x*−*y*, *x*, −*z*; (v) −*x*+*y*, −*x*, *z*; (vi) *x*, *y*, *z*−1; (vii) *x*−*y*, *x*, −*z*+1; (viii) *y*, −*x*+*y*, −*z*+1; (ix) −*x*, −*y*, −*z*+1; (x) *x*, *y*, *z*+1.

### Hydrogen-bond geometry (Å, º)

**Table:**

*D*—H···*A*	*D*—H	H···*A*	*D*···*A*	*D*—H···*A*
C1—H1*A*···I1^vi^	0.98	3.20	4.178 (3)	177
C3—H3*A*···I1^vi^	0.98	3.20	4.178 (3)	174
C4—H4*C*···I1	0.98	3.00	3.967 (3)	170

Symmetry code: (vi) *x*, *y*, *z*−1.
